# Doctors’ life stories in undergraduate medical education: definition, key concepts and uses – a scoping review

**DOI:** 10.1186/s12909-025-07960-8

**Published:** 2025-10-09

**Authors:** James Nixon, Helen West

**Affiliations:** 1 School of Medicine, University of Liverpool, Liverpool, United Kingdom; 2 Department of Psychology, University of Liverpool, Liverpool, United Kingdom

**Keywords:** Narrative medicine, Doctors’ life stories, Undergraduate medical education, Scoping review

## Abstract

**Background:**

Stories are an integral aspect of everyday life, and within medicine and medical education a wide range of stories are told every day. These stories include patients’ stories or stories from those who care for patients. Doctors share stories and do so for many reasons. This includes doctors’ life stories, which we have defined for the purposes of this review as non-fiction stories told first-hand by doctors about their own experiences. When used in undergraduate medical education, these doctors’ life stories allow students to explore, reflect on, and learn from aspects of clinical life they may not yet have experienced. The evidence on doctors’ life stories is sparse however, and this review aimed to explore doctors’ life stories within the context of undergraduate medical education.

**Methods:**

A scoping review methodology was utilised, informed by established methodological guidance and recommendations. A search of five databases, the grey literature, and a hand search of the references of the included articles was carried out. Data from included articles were then collated and analysed using descriptive numerical summary analysis and qualitative content analysis.

**Results:**

A total of 4,978 articles were screened, and 48 articles were included. Included articles were published over a 33-year period, with a significant increase in the number of articles published in the last 10 years. The findings from this review show that doctors’ life stories have been used in multiple key areas of undergraduate medical education and to achieve a variety of intended educational outcomes. The findings highlight a lack of conceptual clarity, evidenced by the range of terms used to describe doctors’ life stories and the lack of a clear definition being described within the literature. To address this, the authors propose a definition to enhance conceptual clarity, complemented by a conceptual framework that provides a structured representation of the phenomenon.

**Conclusion:**

Doctors’ life stories are an emerging topic area within undergraduate medical education and are used in areas that are crucial in supporting the development of medical students into doctors. This scoping review acts as a foundation to guide teaching practice and future research within this area.

**Supplementary Information:**

The online version contains supplementary material available at 10.1186/s12909-025-07960-8.

## Background

Sharing and listening to stories is a vital part of human life [1, 2]. It is through stories that we organise our thoughts, make sense of the world, share experiences and connect with others [1, 3–6]. Within medicine and medical education, a multitude of stories are told every day, with multiple accounts of the use of stories within medicine throughout history [7–10].

Doctors tell stories and do so for many reasons [1, 6, 11]. Doctors’ stories can be fiction or non-fiction; they can be first-hand about their own experiences, or second-hand in which they share patients’ or other healthcare professionals’ stories [11–15]. Doctors’ life stories, which we have defined for the purposes of this review, are non-fiction stories, told first-hand by doctors about their own experiences. Within medical education, these doctors’ life stories allow medical students to explore and reflect on experiences they may not yet have experienced or may not want to experience, and to learn from these experiences [11, 16].

With the advent of Evidence Based Medicine (EBM), medical education moved away from ‘subjective’ stories to ‘objective’ facts and figures [2, 17]. However, critics have suggested that EBM does not fully consider the humanistic aspects of medicine [18, 19]. Alongside this, medical educators and the general public noted concerns that medical care had become depersonalised [20]. This led to increased awareness around person-centred care and an increased emphasis on the Health Humanities within the medical curriculum [19, 20].

Narrative Medicine has been suggested as a complementary approach to EBM that promotes the rehumanisation of medicine [21–24]. Narrative Medicine was defined at an international consensus conference as “a fundamental tool to acquire, comprehend and integrate the different points of view of all the participants having a role in the illness experience.” [1] (p.177). Stories are an integral aspect of Narrative Medicine, and within medicine there are various types of stories [1, 8, 25]. The most prominent type described in the literature is patients’ stories, which offer the experience of “living through and not simply knowledge about” patients [26] (p.48). Alongside patient stories and stories from others who care for patients, doctors’ life stories are thought to contribute to the “rehumanisation” of medicine [8].

While there is a growing body of literature on patients' stories, the evidence on doctors’ life stories is sparse. There are also multiple terms used when discussing doctors’ life stories. It is therefore beneficial to gain a better understanding of what doctors’ life stories are and how they are used. To date, no scoping or systematic reviews on this topic have been published or registered on databases.

This research aimed to explore doctors’ life stories within the context of undergraduate medical education. This involved exploring a broad and complex topic area, which a scoping review methodology was best suited for. This methodology allowed the research to clarify key concepts, definitions and uses of doctors’ life stories in undergraduate medical education, which aligns with the aims of the study. By enhancing our understanding of this topic area and highlighting gaps within the current literature, this research can inform current teaching practice and guide future research.

## Methods

A scoping review methodology, guided by a constructivist research paradigm, was utilised for this study. This paradigm views doctors’ life stories in undergraduate medical education as a construct shaped by the individual experiences and interactions of students, recognising the diverse perspectives and meanings that emerge. The review was conducted by two lecturers: JN is a lecturer in the School of Medicine and a doctor in palliative medicine, and HW is a lecturer in the Department of Psychology. Throughout the review, we engaged in reflexive discussion to acknowledge and critically consider how our professional backgrounds and perspectives may have influenced our approach to the review.

A scoping review methodological framework was devised, guided by the scoping review methodological framework by Arksey and O’Malley and the recommendations by Levac et al. [27, 28]. The Joanna Briggs Institute (JBI) Manual for Evidence Synthesis 2024, specifically Chapter 10: Scoping Reviews, and the Preferred Reporting Items for Systematic reviews and Meta-Analyses (PRISMA) extension for Scoping Reviews (PRISMA-ScR) Checklist were also used to guide the methodological framework [29, 30]. A copy of this methodological framework can be found in Additional File 1.

As recommended by the JBI Manual for Evidence Synthesis, a scoping review protocol was developed and registered on the Open Science Framework registries (10.17605/OSF.IO/Y8BV6).

### Step 1: identifying the review question

This research aimed to explore doctors’ life stories in undergraduate medical education. To do this the following review question was proposed: “How are doctors’ life stories described in the undergraduate medical education literature?”. To answer this, the following sub-questions were devised:

1. How are doctors’ life stories defined in the literature?
2. What are the key concepts of doctors’ life stories in the undergraduate medical education literature?
3. In what ways are doctors’ life stories used in undergraduate medical education?
4. What are the current gaps within the literature?

### Step 2: identifying relevant studies

A search strategy was developed after liaison with the University subject librarian. The search was carried out in three steps, with database searching the first step, the second step consisted of searching the grey literature, and the final step involved hand searching the references of the included articles. No date limitations were placed on the time span prior to the search.

The review aim and questions were considered and the following search terms, along with Boolean operators, were used to search: (doctor* OR physician* OR clinician*) AND (stories OR story OR narrative* or narration* OR storytelling OR “Lived Experience*”) AND (“Undergraduate Medical Education” OR “Medical School” OR “Medical Student*”).

A pilot search was carried out using Medline (OVID), the results of this search were reviewed and the search strategy refined. In collaboration with the University subject librarian, five databases were identified to ensure a broad and comprehensive search of the relevant literature. These databases were Medline (OVID), Web of Science (Core Collection), Scopus, ERIC, and APA PsycINFO. The search strategies for each of the databases can be found in Additional File 2. The grey literature was searched using the web search engine DuckDuckGo, which does not track user data and is therefore less likely to personalise or skew search results based on previous browsing history [31]. In Line with the JBI guidance to conduct as comprehensive a search as possible while considering time and resource constraints, the authors decided to include the first 1000 results from the grey literature search [30].

### Step 3: study selection

Eligibility criteria were developed using the PCC (Population/Participants, Concept, Context) framework (Table 1).

**Table 1:** Eligibility (inclusion/exclusion) criteria

	*Inclusion*	*Exclusion*
Participants	- Medical Students	- Anyone other than medical students
Concept	- Doctors’ life stories	- Stories from perspectives other than doctors (for example patients’, other healthcare professionals’ or students’ stories) - Doctors’ stories which are not first-hand (e.g. doctors discussing patients’ stories) - Fictional stories
Context	- Undergraduate Medical Education	- Any context other than Undergraduate Medical Education
General Criteria	- Published at any time prior to the final search - All geographical areas - Available in English (either written in English or available in a translated format) - Peer reviewed and non-peer reviewed literature including primary research, reviews, commentaries and opinion papers, and grey literature	- Published after the final search - Not available in English

Articles from the database search were collated and managed using Endnote 21, duplicate articles were removed, and the remaining articles were screened. All articles retrieved from the grey literature search and a hand search of the references of the included articles (citation search) were screened. Screening was carried out by JN using a two-stage process, with the first stage involving screening the titles and abstracts of the articles against the eligibility criteria. Included articles then proceeded to the second stage, which assessed the articles’ full text against the eligibility criteria. Articles included after both stages then proceeded to data charting and analysis. A second check was carried out by HW in which a sample of 10% of the articles were collated and screened for each stage. Rayyan.ai was used for this second check as it allowed both screeners to remain blinded until screening was complete [32]. Prior to the second check, an aim of > 90% agreement between screeners was agreed. If > 90% agreement was not achieved, both screeners would discuss the disagreements to reach a consensus. If a consensus could not be reached, then a third screener would be consulted to make a final decision. For both stages of the screening process, > 90% agreement between JN and HW was met. During the screening process the results were collated on a PRISMA flowchart which is presented in the results section of this paper.

### Step 4: charting the data

A data charting form and data charting guidance were developed to collate and summarise the data from the included articles. This data charting form was piloted against a sample of 25% of the included articles and then refined. The completed data charting form can be found in Additional File 3, and the data charting guidance in Additional File 4.

### Step 5: collating, summarising and reporting the results

The data from this review was then collated and analysed using descriptive numerical summary analysis and qualitative content analysis. The results of this analysis are summarised and discussed in the following sections.

## Results

**Fig. 1:**
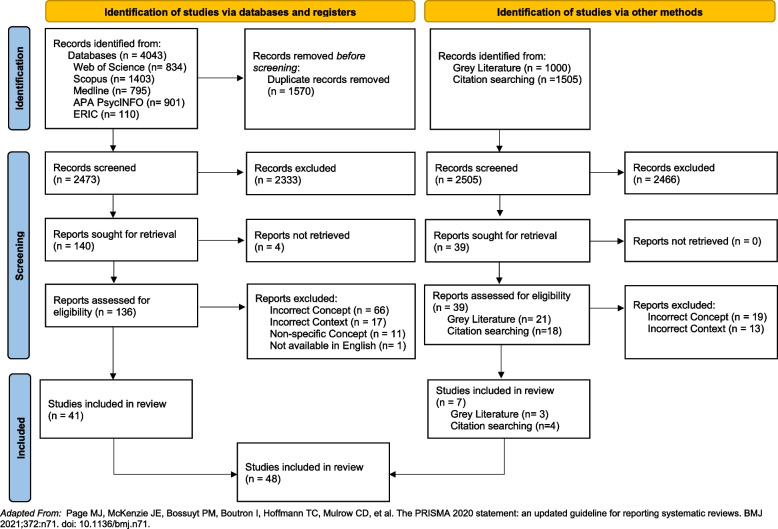
PRISMA 2020 flow diagram

A full search of the databases, grey literature and the included articles’ references (citation search) returned 6,548 articles. The database search identified 4043, the grey literature search included 1000, and the citation search included 1505. After removing duplicate articles from the database search, 4978 articles were screened, and 179 articles’ full text were sought for retrieval. After an extensive search by both authors and the University Librarians, the full text of four articles were unable to be retrieved. The remaining 175 articles’ full text were assessed against the eligibility criteria and 48 articles were included within this review. The included articles consisted of 41 identified from the database search, 3 from the grey literature search, and 4 from the citation search. The articles identified from the database search included 24 identified through multiple databases and 17 identified from one database (7 articles PsycINFO, 5 articles Web of Science, 4 articles Scopus, 1 article Medline). A PRISMA flow diagram of this process can be found in Fig. 1.

### Publication characteristics

Included articles were published over a 33-year period from 1992–2024, with half of these articles originating from the United States of America. The types of articles were placed into three categories with the predominant category being research articles (16 articles). Other categories included innovation articles/case reports (15 articles), and commentaries/review articles (12 articles). Articles that did not fit these classifications were grouped under ‘Other’ and comprised two book chapters, one conference paper, one PhD thesis, and one webpage. A summary of the publication characteristics of the included articles can be found in Table 2.

**Table 2:** Publication characteristics

	Number of Articles	Article Reference(s)
***Year:***		
2014–2024	32	[12, 15, 33–62]
2003–2013	10	[63–72]
1992–2002	6	[73–78]
***Location:***		
United States of America	24	[15, 34, 35, 37, 41, 44, 45, 53–55, 60, 61, 63, 65–68, 71–73, 75–78]
United Kingdom	7	[33, 39, 40, 46–48, 50]
Canada	7	[36, 56, 58, 59, 62, 70, 74]
Australia	2	[51, 52]
India	2	[38, 42]
Israel	2	[43, 49]
France	1	[57]
Greece	1	[69]
Taiwan	1	[64]
Not Stated	1	[12]
***Type of Article:***		
Research Article	16	[33, 36, 39, 42, 43, 47–49, 57–59, 61, 62, 67, 70, 71]
Innovation Article/Case Report	15	[34, 38, 45, 50–56, 63–65, 73, 74]
Commentary/Review Article	12	[12, 15, 40, 44, 60, 66, 69, 72, 75–78]
Other*	5	[35, 37, 41, 46, 68]

^*^Includes 2 book chapters, 1 conference paper, 1 PhD thesis and 1 webpage

During this scoping review, three main themes were identified from the results: Learning Activity, Design and Delivery, and Intended Outcomes. These will be discussed further below.

### Learning activity—what doctors’ life stories are

The Learning Activity theme outlines how articles describe doctors’ life stories, including the terminology, definition, characteristics and aspects. As shown in Table 3, 30 different terms were used to describe doctors’ life stories in the included articles. Only 9 articles used one term while the remaining articles used between 2–5 different terms interchangeably. The most common term was “narrative” with 65% of included articles using this term (31 articles), followed by “story” with 52% (25 articles). A definition of doctors’ life stories or the associated term(s) used within the article was provided by only two articles, while an overarching definition for story and/or narrative was provided by four articles. These definitions can be found in Table 4.

**Table 3:** Terminology

	No. of Articles	Article Reference(s)
Narrative	31	[12, 15, 33, 34, 36–39, 41, 42, 51, 52, 54–56, 58–61, 63, 65–69, 71, 72, 74–76, 78]
Story	25	[12, 33, 35, 36, 39–41, 44, 47, 48, 51, 57–60, 62, 66, 68, 69, 71, 73, 74, 76–78]
Storytelling	11	[12, 33, 35, 41, 45, 50, 56, 57, 60, 62, 77]
Personal Story	6	[45, 50, 54, 55, 58, 72]
Lived Experience	4	[43, 46, 56, 59]
Experience	3	[40, 42, 53]
Anecdote	2	[12, 47]
Life Story	2	[64, 77]
Biography	1	[69]
Case	1	[48]
Case Study	1	[42]
Case Vignette	1	[42]
Clinical Narrative	1	[37]
Dialogue	1	[35]
Doctor's Account of Practice	1	[67]
History	1	[49]
Life Narrative	1	[64]
Living Experience	1	[49]
Narrative Storytelling	1	[71]
Personal Anecdote	1	[54]
Personal Autobiography	1	[39]
Personal Experience	1	[47]
Personal Illness Story	1	[59]
Personal Testimony	1	[46]
Real Case	1	[70]
Real-Life Experience	1	[12]
Reflective Narrative	1	[70]
Self-disclosed History	1	[43]
Shared Physician Narrative	1	[53]
Testimony	1	[57]

**Table 4:** Definitions of doctors’ life story, story and narrative

Doctors’ Life Stories:
• "the employment of narratives, anecdotes, and real-life experiences to stories to share and teach medical concepts and principles." [12]
• "the big things … of the physician's life—the great unmentionables that are yet everyday aspects of doctoring" [77]
Story:
• “a detailed, character-based narration of a character's struggles to overcome obstacles and reach an important goal.” [39]
• “a form of authentication and of being listened to.” [40]
Narrative:
• “a story that includes a plot, characters, a setting and a theme. A narrative supplies and reveals the themes by which we seek to unify the temporal, historical dimension of our existence.” [68]
• “a sequence of events connected together in a way that gives them meaning.” [39]
• “means more than content or story. It involves a style of presentation.” [75]

The articles’ text was screened for descriptions of the characteristics and aspects of doctors’ life stories. The characteristics included the descriptors used for doctors’ life stories, for example “believable” and “focused”. The aspects included the features described that make a doctors’ life story, for example “experiences and reflections of life in medical practice”. Aspects were described in 14 articles, characteristics in 5, and both characteristics and aspects were described in 4 articles. The characteristics and aspects described were separately analysed, with 6 categories developed for characteristics and 2 categories for aspects (Table 5).

**Table 5:** Characteristics and aspects

***Characteristics***	*Article Reference(s)*
Believable	[39]
Focused	[74, 77]
Thought-provoking and/or Role-modelling	[39, 69]
Memorable	[39]
Personal	[74, 77]
Subjective	[75]
***Aspects***	
Experiences and reflections of life in medical practice	[12, 15, 37, 39, 48, 50, 53, 54, 64, 73–75]
Important specific message	[72, 77]

### Design and delivery – how they are used

The Design and Delivery theme outlines how the articles structure and deliver doctors’ life stories within undergraduate medical education. A summary of these results can be found in Table 6.

**Table 6:** Design and delivery

	Number of Articles	Article References
***Setting:***		
Medical School	23	[34, 37–40, 43, 45–49, 52–55, 59, 60, 65, 67, 69–71, 73]
Online	7	[36, 56–58, 60–62]
External Location	3	[41, 58, 63]
Clinical Setting	2	[35, 66]
Not stated	15	[12, 15, 33, 42, 44, 50, 51, 64, 68, 72, 74–78]
***Attendance Requirement:***		
Voluntary	20	[34, 36, 38, 40, 41, 46, 48, 50, 55–64, 67, 73]
Compulsory	8	[39, 45, 49, 51–54, 65]
Not Stated	20	[12, 15, 33, 35, 37, 42–44, 47, 66, 68–72, 74–78]
***Topic Areas:***		
Wellbeing	12	[40, 43–47, 49, 54, 55, 57, 59, 60]
Professionalism	9	[42, 47, 53, 70–72, 74, 75, 77]
Health Humanities	5	[15, 38, 51, 65, 69]
Professional Identity Formation	5	[34, 52, 53, 73, 75]
Narrative Medicine	5	[12, 39, 41, 67, 78]
Specialty Specific*	5	[36, 58, 61, 63, 66]
Reflection	3	[37, 48, 64]
Patient Safety	2	[33, 54]
Empathy	2	[62, 68]
Equality, Diversity and Inclusion	2	[35, 56]
Medical Ethics	1	[76]
Leadership and Management	1	[50]
***Format:***		
Live	22	[35, 39–41, 43, 45–49, 51–55, 58, 63, 64, 66, 70, 71, 77]
Written/Graphic	12	[15, 34, 38, 51, 63, 67, 68, 72–76]
Recorded	6	[36, 54, 56, 57, 61, 62]
Animation	1	[33]
Not Stated	10	[12, 37, 42, 44, 50, 59, 60, 65, 69, 78]
***Associated Elements:***		
Reflection	17	[12, 34, 38, 45, 51–54, 57, 60, 63, 64, 67, 68, 71, 73, 77]
Group Discussion	15	[33, 40, 43, 47–49, 53, 58, 63, 65–68, 74, 77]
Sharing own story	5	[37, 45, 54, 60, 71]
Creating own story	1	[34]
Not Stated	20	[15, 35, 36, 39, 41, 42, 44, 46, 50, 55, 56, 59, 61, 62, 69, 70, 72, 75, 76, 78]

^*^Includes Rural Medicine, Psychiatry and Palliative Medicine

The setting was described in 33 articles, with the medical school being the most frequently described (23 articles). Other settings included an online setting (7 articles), an external location including retreats or events venues (3 articles), and the clinical setting (2 articles). Two articles described multiple settings. Attendance requirements were described in 28 articles, with voluntary attendance being the most common (20 articles). Participants included medical students from all years of the medical degree. Six articles described a mixed group of participants including medical students, doctors, other healthcare professionals, and/or other healthcare students.

The included articles’ text was analysed to identify the topic areas that doctors’ life stories were used in. From this, 12 categories were identified, with Wellbeing being the most common topic area (12 articles). The format of the doctors’ life stories were placed into four categories, with 10 articles not stating a format and eight articles describing multiple formats. The most common format was live, which included live spoken in-person and/or online (22 articles), followed by written/graphic, which included written prose, written poetry and/or graphics (12 articles). Other formats included recorded, which included recorded audio and/or video (6 articles) and animation (1 article). Twenty-eight articles described an associated element that was used alongside doctors’ life stories. The most common associated element was reflection (17 articles), followed by group discussion (15 articles). Ten articles had multiple associated elements, with five of these articles describing both reflection and group discussion.

### Intended outcomes – what they aim to achieve

The Intended Outcomes theme outlines the educational outcomes that the authors of the included articles sought to achieve or suggested could be achieved through the use of doctors’ life stories in undergraduate medical education. The included articles’ text was analysed for intended outcomes and nine categories were developed (Table 7). All the included articles were placed within categories, with 32 articles assigned to multiple categories as they described multiple intended outcomes. The most commonly described intended outcome was promoting reflection (19 articles). This was followed by enhancing professional development (16 articles), which encompassed enhancing professional values and behaviours as well as enhancing the development of professional skills.

**Table 7:** Intended outcomes

	Number of Articles	References
Promote reflection	19	[12, 15, 37, 39, 41, 47, 48, 51, 53, 56, 62–64, 68, 71, 72, 74, 77, 78]
Enhance professional development	16	[34, 39, 42, 45, 51, 59, 62–65, 67–69, 72, 74, 75]
Change attitudes, perceptions and beliefs	10	[33, 43, 44, 46, 49, 55, 57, 58, 61, 62]
Promote professional identity formation	9	[15, 34, 39, 52, 53, 56, 59, 65, 73]
Create meaning and build knowledge	9	[33, 36, 39, 50, 57, 61–63, 74]
Create a sense of community	9	[35, 40, 41, 55, 56, 59, 60, 62, 67]
Promote self-care, compassion and wellbeing	7	[40, 45, 51, 59, 60, 64, 76]
Promote a better understanding of the human side of healthcare	6	[12, 34, 41, 62, 68, 69]
Enhance cultural, social and ethical awareness	6	[12, 35, 38, 56, 58, 76]

## Discussion

This scoping review explored doctors’ life stories in undergraduate medical education, providing a novel synthesis of the evidence within this topic area. The results of this review highlight that this is an emerging topic area evidenced by the increase in the number of articles published in the last 10 years.

### Definition of doctors’ life stories

Prior to considering the definition, it was important to consider the terminology being used. Within the included articles there were 30 different terms used to describe doctors’ life stories, with some terms being used interchangeably within the same article. While a large proportion of the articles used the terms narrative and/or story, this was expected as these terms were used as part of the search strategy.

This concept proliferation, in which multiple terms are used, may be due to a lack of conceptual clarity surrounding doctors’ life stories. This lack of conceptual clarity is amplified by the lack of a definition of doctors’ life stories being described within the literature. To enhance conceptual clarity surrounding doctors’ life stories, the definitions described within the literature alongside the characteristics and aspects highlighted during this scoping review were considered and a definition proposed. This definition is “Doctors’ life stories are personal, focused, non-fiction stories about doctors’ experiences and reflections of life in medical practice, told first-hand to provide insight into life as a doctor”. As further literature on the topic is published, this definition can be further refined.

### Key concepts

Alongside considering the definition, it is also important to identify the key concepts of the phenomenon “doctors’ life stories in undergraduate medical education”. While the results of this review identified a number of concepts, not all concepts were seen as integral or key. By analysing these concepts and considering their role within the phenomenon, five key concepts were identified (Table 8).

**Table 8:** Key concepts of doctors’ life stories

*Key Concept*	*Description*
Doctors’ Life Stories	The elements that make up a doctors’ life story including its definition, characteristics and aspects
Curriculum Area	The topic area(s) in which the doctors’ life stories are used
Delivery Methods	The ways in which doctors’ life stories are delivered including the format and the associated elements used alongside
Learning Environment	The setting in which doctors’ life stories are used
Potential Educational Impact	The ways in which doctors’ life stories may contribute to students’ learning and development

**Fig. 2:**
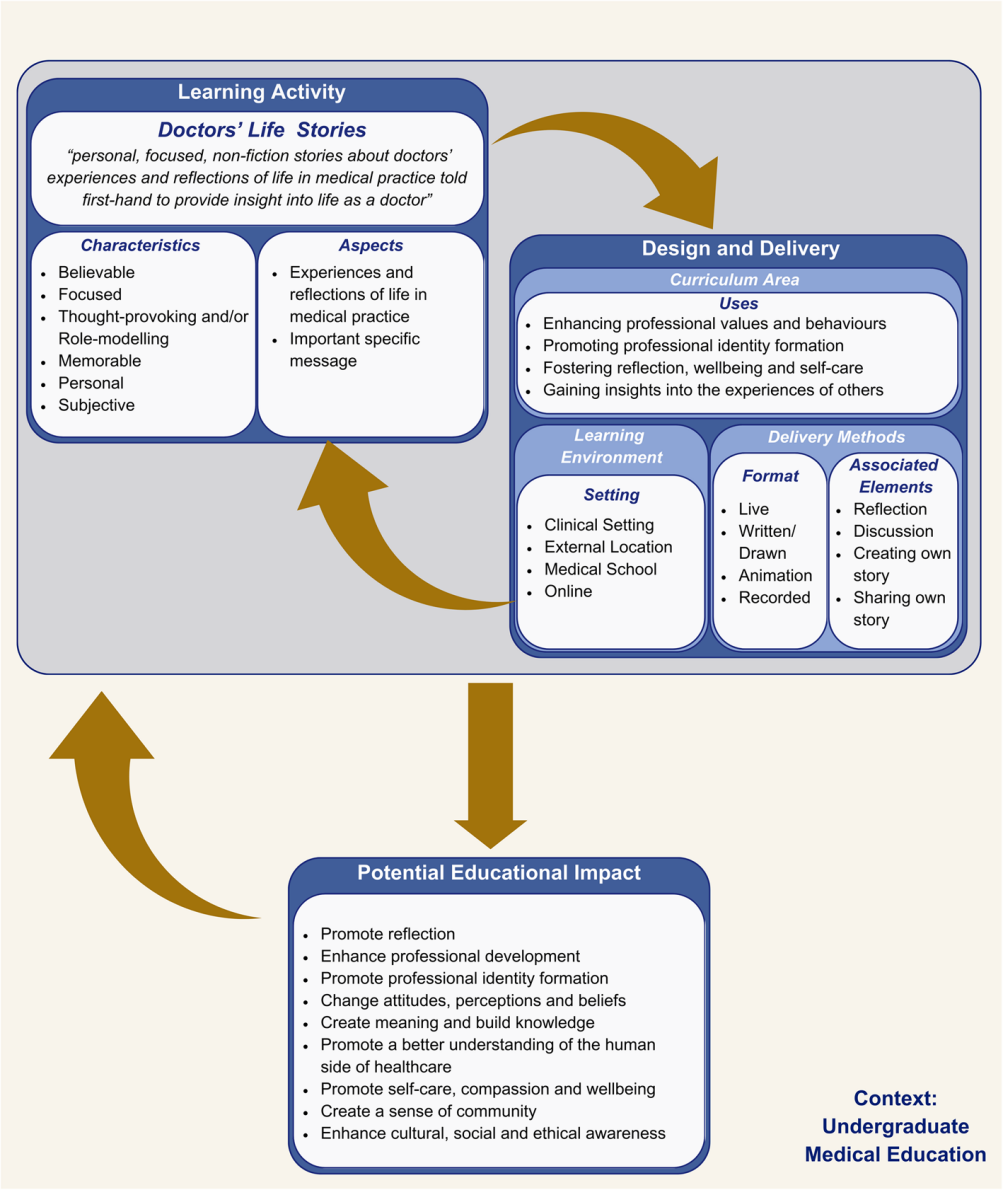
Conceptual Framework of the phenomenon “Doctors’ Life Stories in undergraduate medical education”

The interaction between these key concepts was also considered. This allowed the composition of a conceptual framework (Fig. 2), which was composed with a constructivist lens, in which knowledge is constructed through individual student’s experiences and interactions. Prior knowledge and experience also provide a foundation, shaping how students learn. Given this, the framework acknowledges that educational outcomes will vary, shaped by each student’s personal experiences and interactions with others.

The conceptual framework consists of three interrelated components: Learning Activity (Doctors’ Life Stories), Design and Delivery, and Potential Educational Impact. The Learning Activity defines the core attributes of doctors’ life stories, including their definition, characteristics, and aspects. The Design and Delivery component outlines how the Learning Activity is integrated and delivered, including the curriculum area (topic area), delivery methods (format and associated elements), and the learning environment (setting). The Learning Activity and Design and Delivery components interact dynamically and, when aligned, may lead to a Potential Educational Impact. This Potential Educational Impact also feeds back into the Learning Activity and the Design and Delivery in which it can influence these components.

This conceptual framework provides a structured representation of the phenomenon, enhancing clarity and outlining the key components and their interactions. Further research would facilitate the testing and refinement of this framework.

### Uses

The results of this review demonstrate that doctors’ life stories have numerous uses within undergraduate medical education. After analysing the intended outcomes (Table 7) and topic areas in which these stories have been used (Table 6), four main categories of ways doctors’ life stories could contribute to undergraduate medical education were identified, as shown below:

1. Enhancing professional values and behaviours, which are the essential qualities required to be a doctor as highlighted by the General Medical Council in the UK and CanMEDs in Canada [79, 80].
2. Promoting professional identity formation, which is the critical transition or transformation from lay person to doctor [81, 82].
3. Fostering reflection, wellbeing and self-care, which can support emotional resilience, mitigate burnout and promote professional development.
4. Gaining insights into the experiences of others, which can create a sense of community, broaden perspectives, and enhance cultural, social and ethical awareness.

These areas are integral to undergraduate medical education and play crucial roles in shaping medical students’ development into competent doctors. This review highlights these key uses, providing an opportunity for exploration of the various ways that they can be integrated into the medical curriculum.

### Gaps within the literature

By mapping the literature surrounding doctors’ life stories in undergraduate medical education, this review identified a number of gaps within the current literature. As previously noted in this discussion there is a current ambiguity in the literature surrounding this phenomenon. While this review aimed to address the lack of detail surrounding the definition and key concepts, further detail surrounding the processes by which doctors’ life stories are selected and created, as well as how they are integrated into the curriculum, would be beneficial.

The articles included in this review also predominantly looked at the formal use of doctors’ life stories, however, further research looking at their informal use would also be beneficial. Our understanding of both the formal and informal use of doctors’ life stories could be enhanced by further research exploring the perceptions and experiences of key stakeholders in medical education, including students, doctors, other healthcare professionals and educators.

### Limitations of this scoping review

This scoping review carried out a broad search of the literature, including a search of five relevant databases and a search of the grey literature. Only articles that were available in the English language were included, which may restrict the results to Western practice and limit their transfer beyond these regions. The presence of concept proliferation, in which multiple terms were used for the concept of doctors’ life stories, may also mean that some articles were not identified as the terms they used were not included in the search strategy.

An established scoping review methodology was used with one author carrying out the full screening and a second author checking a sample of the articles. While this is accepted practice, there is still a risk that some articles may have been missed during screening and therefore not included in this review.

## Conclusion

Doctors’ life stories play a vital role in undergraduate medical education. The results of this review highlight that this is an emerging field, with doctors’ life stories being used in multiple different areas within undergraduate medical education. These areas include enhancing professional values and behaviours and promoting professional identity formation, which are crucial in supporting the development of medical students into future doctors. There is currently a lack of clarity surrounding doctors’ life stories in undergraduate medical education however, and this review aimed to enhance this understanding by proposing a definition for doctors’ life stories and a conceptual framework for this phenomenon.

The findings of this review enhance our understanding of doctors’ life stories in undergraduate medical education, which provides educators with more evidence to evaluate the potential role of doctors’ life stories within their curriculum. The results also provide a foundation for research within this emerging field and highlight the current gaps within the literature, thereby guiding future research and ultimately advancing the field.

## Supplementary Information

Additional file1. Scoping Review Methodological Framework. A table documenting the Scoping Review Methodological Framework used including the five steps taken as part of the scoping review

Additional file2. Individual Database Search Strategies. Description of data: A table documenting the search strategies used for each of the databases searched as part of the scoping review

Additional file3. Completed Data Charting Form. Description of data: A table containing the data charted from the articles included in this scoping review

Additional file4. Data Charting Guidance Form. Description of data: A table documenting the guidance used when carrying out data charting as part of this scoping review

## Data Availability

No datasets were generated or analysed during the current study.

## Abbreviations

EBM: Evidence based medicine
JBI: Joanna Briggs Institute
PRISMA: Preferred reporting items for systematic reviews and meta-Analyses
PRISMA-ScR: Preferred reporting items for systematic reviews and meta-analyses extension for scoping reviews
PCC: Population/participants, concept, context
JN: James Nixon, author
HW: Helen West, author
