# Supplementary material for: Doctors’ life stories in undergraduate medical education: definition, key concepts and uses – a scoping review
Source: BMC Med Educ. 2025 Oct 9;25:1390. doi: 10.1186/s12909-025-07960-8 (PMC12512262; doi:10.1186/s12909-025-07960-8)
Supplement: Supplementary file 1 — Additional file1. Scoping Review Methodological Framework. A table documenting the Scoping Review Methodological Framework used including the five steps taken as part of the scoping review [file 12909_2025_7960_MOESM1_ESM.docx]

# Additional File 1: Scoping Review Methodological Framework

| Step 1: Identifying the review question | - Identify a review question to provide a broad scope (27, 28). - Define the scope of inquiry (concept, target population and outcomes of interest) (28, 30) - Define intended outcomes of the study (28, 30) - Explain rationale for completing the study (27, 28, 30) |
| --- | --- |
| Step 2: Identifying relevant studies (in liaison with subject librarian) | - Develop a search strategy (27, 28, 30) - Carry out a pilot search (30) - Review results of pilot search and make amendments to search strategy and review question (if necessary) (30) - Decide information sources to search (27, 30) - Consider search limitations; for example time span and language (27) - Carry out full search and document results (27, 28, 30) |
| Step 3: Study selection | - Develop inclusion/exclusion criteria (27, 28, 30) - Carry out screening through a two-stage process (27, 28, 30)   - First Screen: Abstract/Title Screen   - Second Screen: Full Text screen - Collate and present results of screening (including a PRISMA flowchart) (29, 30) |
| Step 4: Charting the data | - Develop a data charting form including variables to chart. (27, 28, 30) - Pilot chart against sample of articles and review (28, 30) - Review results of pilot charting exercise and make amendments to data charting form (if necessary) (28, 30) - Carry out data charting (27, 28, 30) |
| Step 5: Collating, summarising and reporting the results | - Consider approach to data analysis (27, 28, 30) - Carry out data analysis (27, 28, 30) - Report the results and produce the outcome that refers to the research objective (27-30) - Discuss the implications for future research, practice and policy. (28) |
