# Supplementary material for: Doctors’ life stories in undergraduate medical education: definition, key concepts and uses – a scoping review
Source: BMC Med Educ. 2025 Oct 9;25:1390. doi: 10.1186/s12909-025-07960-8 (PMC12512262; doi:10.1186/s12909-025-07960-8)
Supplement: Supplementary file 2 — Additional file2. Individual Database Search Strategies. Description of data: A table documenting the search strategies used for each of the databases searched as part of the scoping review [file 12909_2025_7960_MOESM2_ESM.docx]

# Additional File 2: Individual Database Search Strategies

| Database | Search Strategy | Date of Final Search |
| --- | --- | --- |
| Scopus | TITLE-ABS-KEY ( doctor* OR physician* OR clinician* AND stories OR story OR narrative* OR narration* OR storytelling OR "LIVED EXPERIENCE*" AND "UNDERGRADUATE MEDICAL EDUCATION" OR "MEDICAL SCHOOL" OR "MEDICAL STUDENT*" ) | 8^th^ April 2025 |
| APA PsycINFO | ( doctor* OR physician* OR clinician* ) AND ( Stories OR Story OR Narrative* OR Narration* OR Storytelling OR "Lived Experience*" ) AND ( "Undergraduate Medical Education" OR "Medical School" OR "Medical Student*" ) |  |
| ERIC | ( Doctor* OR Physician* OR Clinician ) AND ( Stories OR Story OR Narrative* OR Narration* OR Storytelling OR "Lived Experience*" ) AND ( "Undergraduate Medical Education" OR "Medical School" OR "Medical Student*" ) |  |
| Web of Science | DOCTOR* OR PHYSICIAN* OR CLINICIAN* (Topic) and STORY OR STORIES OR NARRATIVE* OR NARRATION* OR STORYTELLING OR "LIVED EXPERIENCE*"  (Topic) and "Undergraduate Medical Education" OR "Medical Student*" OR "Medical School" (Topic) |  |

| Database | Search Strategy | | Date of Final Search |
| --- | --- | --- | --- |
| Medline (OVID) | 1 | Doctor*.mp. | 8^th^ April 2025 |
|  | 2 | Physician*.mp. |  |
|  | 3 | Clinician*.mp. |  |
|  | 4 | 1 or 2 or 3 |  |
|  | 5 | Stories.mp. |  |
|  | 6 | Story.mp. |  |
|  | 7 | Narrative*.mp. |  |
|  | 8 | Narration*.mp. |  |
|  | 9 | Storytelling.mp. |  |
|  | 10 | "Lived Experience*".mp. |  |
|  | 11 | 5 or 6 or 7 or 8 or 9 or 10 |  |
|  | 12 | Education, Medical, Undergraduate/ |  |
|  | 13 | "Medical School".mp. or Schools, Medical/ |  |
|  | 14 | "Medical Student".mp. or Students, Medical/ |  |
|  | 15 | 12 or 13 or 14 |  |
|  | 16 | 4 and 11 and 15 |  |
