# Supplementary material for: Doctors’ life stories in undergraduate medical education: definition, key concepts and uses – a scoping review
Source: BMC Med Educ. 2025 Oct 9;25:1390. doi: 10.1186/s12909-025-07960-8 (PMC12512262; doi:10.1186/s12909-025-07960-8)
Supplement: Supplementary file 3 — Additional file3. Completed Data Charting Form. Description of data: A table containing the data charted from the articles included in this scoping review [file 12909_2025_7960_MOESM3_ESM.docx]

# Additional File 3: Completed Data Charting Form

| **Title** | **Publication Characteristics** | **Terminology** | **Methods** | **Intervention** |
| --- | --- | --- | --- | --- |
| "A Life in Medicine": Stories from a Dartmouth Medical School Elective^70^ | **Author:** M Moore-West, RM Testa and J O'Donnell | **Terminology Used:** Story | **Setting:** Medical School | **Format:** Written Prose |
|  | **Year:** 1998 | **Definition:** None given | **Attendance Requirement:** Voluntary | **Associated Elements:** Reflection |
|  | **Journal:** Academic Medicine | **Characteristics:** Not stated | **Topic Area:** Professional Identity Formation | **Intended Outcome:** Promote Professional Identity Formation |
|  | **Type of Article:** Innovation article/Case report | **Aspects:** Experiences of being a doctor and working as a doctor. | **Participant Characteristics:** Fourth Year Medical Students | **Type of doctor:** Various, non-specific |
|  | **Location:** USA |  |  |  |
| A podcast to teach medical humanities at medical school: a text-mining study of students’ lived experiences^54^ | **Author:** E Roze et al. | **Terminology Used:** Storytelling, testimony, story | **Setting:** Online | **Format:** Recorded spoken audio (podcast) |
|  | **Year:** 2024 | **Definition:** None given | **Attendance Requirement:** Voluntary | **Associated Elements:**  Reflection |
|  | **Journal:** Medical Education Online | **Characteristics:** Not stated | **Topic Area:** Wellbeing- Mental health | **Intended Outcome:** Create meaning and build knowledge; Change attitudes, perceptions and beliefs |
|  | **Type of Article:** Research Article | **Aspects:** Not stated | **Participant Characteristics:**  Medical Students (non-specific) | **Type of doctor:**  Not stated |
|  | **Location:** France |  |  |  |
| Animated stories of medical error as a means of teaching undergraduates patient safety: an evaluation study^29^ | **Author:** K Cooper, E Hatfield and J Yeomans | **Terminology Used:** Storytelling, story, narrative | **Setting:** Not Stated | **Format:** Animated Video (with recorded audio) |
|  | **Year:** 2019 | **Definition:** None given | **Attendance Requirement:** Not Stated | **Associated Elements:**  Group Discussion |
|  | **Journal:** Perspectives on Medical Education | **Characteristics:** Not stated | **Topic Area:** Patient safety- medical error | **Intended Outcome:** Change attitudes, perceptions and beliefs; Create meaning and build knowledge |
|  | **Type of Article:** Research Article | **Aspects:** Not stated | **Participant Characteristics:**  Final year medical students | **Type of doctor:**  Junior Doctors |
|  | **Location:** UK |  |  |  |
| Challenging Perceptions about rural practice using narratives: a living library approach in medical education^55^ | **Author:** G Perez et al. | **Terminology Used:** Narrative, personal experience, story, personal story | **Setting:** Online, External venue | **Format:** Live spoken, in-person |
|  | **Year:** 2024 | **Definition:** None given | **Attendance Requirement:** Voluntary | **Associated Elements:** Group discussion |
|  | **Journal:** Frontiers in Medicine | **Characteristics:** Not stated | **Topic Area:** Specialty specific – rural medicine | **Intended Outcome:**  Enhance cultural, social and ethical awareness; Change attitudes, perceptions and beliefs |
|  | **Type of Article:** Research Article | **Aspects:** Not stated | **Participant Characteristics:**  Medical Students (non-specific) | **Type of doctor:** Doctors working in rural medicine |
|  | **Location:** Canada |  |  |  |
| Comics and Medicine: Peering Into the Process of Professional Identity Formation^30^ | **Author:** M Green | **Terminology Used:** Narrative | **Setting:** Medical School | **Format:** Graphic Novels |
|  | **Year:** 2015 | **Definition:** None given | **Attendance Requirement:** Voluntary | **Associated Elements:** Reflection; Creating own story |
|  | **Journal:** Academic Medicine | **Characteristics:** Not stated | **Topic Area:** Professional Identity Formation | **Intended Outcome:**  Promote Professional Identity Formation; Promote a better understanding of the human side of healthcare |
|  | **Type of Article:** Innovation article/Case report | **Aspects:** Not stated | **Participant Characteristics:**  Fourth and fifth year medical students | **Type of doctor:** Various, non-specific |
|  | **Location:** USA |  |  |  |
| Country Doctors in Literature: Helping Medical Students Understand What Rural Practice is All About^60^ | **Author:** J Shapiro, R Longenecker | **Terminology Used:**  Narrative | **Setting:** External location | **Format:** Live spoken, in-person; Written prose |
|  | **Year:** 2005 | **Definition:** None given | **Attendance Requirement:** Voluntary | **Associated Elements:** Group Discussion; Reflection |
|  | **Journal:** Academic Medicine | **Characteristics:** Not stated | **Topic Area:** Specialty specific- Rural Medicine | **Intended Outcome:** Create meaning and build knowledge; Promote Reflection |
|  | **Type of Article:** Innovation article/Case report | **Aspects:** Not stated | **Participant Characteristics:**  Medical Students in years 1-4. | **Type of doctor:** Doctors who work within rural practice |
|  | **Location:** USA |  |  |  |
| Diversity Dialogue: An innovative model for diversity training^31^ | **Author:**  E Sharon, A Emmerich, R Parekh. R Parkh (Editor) | **Terminology Used:** Story, Dialogue, Storytelling | **Setting:** Clinical Setting | **Format:** Live spoken, in-person |
|  | **Year:** 2014 | **Definition:** None given | **Attendance Requirement:**  Not Stated | **Associated Elements:** Not Stated |
|  | **Journal:** Springer Publishing | **Characteristics:** Not stated | **Topic Area:** Equality, Diversity, Inclusivity | **Intended Outcome:** Create a sense of community; Enhance cultural, social and ethical awareness. |
|  | **Type of Article:** Other (Book Chapter) | **Aspects:** Not stated | **Participant Characteristics:**  Not Stated | **Type of doctor:** Not Stated |
|  | **Location:** USA |  |  |  |
| Enhancing Psychiatry Education through Podcasting: Learning from the Listener Experience^32^ | **Author:** S Hanafi et al. | **Terminology Used:**  Story, narrative | **Setting:** Online | **Format:** Recorded spoken audio |
|  | **Year:** 2022 | **Definition:** None given | **Attendance Requirement:** Voluntary | **Associated Elements:** None |
|  | **Journal:** Academic Psychiatry | **Characteristics:** Not stated | **Topic Area:** Specialty specific- Psychiatry | **Intended Outcome:** Create meaning and build knowledge |
|  | **Type of Article:** Research Article | **Aspects:** Not stated | **Participant Characteristics:**  Mixed (UG/PG students, qualified health professionals) | **Type of doctor:** Psychiatrists |
|  | **Location:** Canada |  |  |  |
| Ethics and teaching mindfulness to physicians and healthcare professionals^33^ | **Author:** M Krasner, P Luck. L Monteiro, J Compson, F Musten (Editors) | **Terminology Used:** Narrative, clinical narrative | **Setting:** Medical School | **Format:** Not stated |
|  | **Year:** 2017 | **Definition:** None given | **Attendance Requirement:**  Not stated | **Associated Elements:** Sharing own story |
|  | **Journal:** Springer Publishing | **Characteristics:** Not stated | **Topic Area:** Reflection- Mindfulness | **Intended Outcome:** Promote reflection |
|  | **Type of Article:** Other (Book Chapter) | **Aspects:** Personal experiences | **Participant Characteristics:**  Third Year medical students | **Type of doctor:** Not stated |
|  | **Location:** USA |  |  |  |
| Evaluating the Dear MD to be Podcast as an Equity, Diversity and Inclusion Resource: a cross-sectional survey analysis^53^ | **Author:** I Zera Khermani et al. | **Terminology Used:** Lived experience, narrative, storytelling | **Setting:** Online | **Format:** Recorded spoken audio (podcast) |
|  | **Year:** 2024 | **Definition:** None given | **Attendance Requirement:** Voluntary | **Associated Elements:** Not Stated |
|  | **Journal:** Canadian Medical Education Journal | **Characteristics:** Not stated | **Topic Area:** Equality, Diversity, Inclusion | **Intended Outcome:** Create a sense of community; Enhance cultural, social and ethical awareness; Promote Professional Identity Formation; Promote Reflection. |
|  | **Type of Article:** Innovation article/Case Report | **Aspects:** Not stated | **Participant Characteristics:** Medical Students (non-specific) | **Type of doctor:** Residents and other healthcare staff |
|  | **Location:** Canada |  |  |  |
| Evaluation of Two Videos that Apply Evidence-Based Strategies to Increase Self-Efficacy and Reduce Opioid-Related Stigma Among Medical Students^58^ | **Author:** E Eschliman et al. | **Terminology Used:** Narrative, real life experiences | **Setting:** Online | **Format:** Recorded spoken video |
|  | **Year:** 2024 | **Definition:** None given | **Attendance Requirement:** Voluntary | **Associated Elements:** Not Stated |
|  | **Journal:** Academic Psychiatry | **Characteristics:** Not stated | **Topic Area:** Specialty specific – psychiatry | **Intended Outcome:** Change attitudes, perceptions and beliefs; Create meaning and build knowledge |
|  | **Type of Article:** Research Article | **Aspects:** Not stated | **Participant Characteristics:** Medical Students (non-specific) | **Type of doctor:** Physicians who treat (or have treated) patients who use opioids |
|  | **Location:** USA |  |  |  |
| Evoking the Moral Imagination: Using Stories to Teach Ethics and Professionalism to Nursing, Medical, and Law Students^71^ | **Author:** M Weisberg, J Duffin | **Terminology Used:** Story, narrative | **Setting:** Not Stated | **Format:** Written prose |
|  | **Year:** 1995 | **Definition:** None given | **Attendance Requirement:**  Not Stated | **Associated Elements:**  Group Discussion |
|  | **Journal:** The Journal of Medical Humanities | **Characteristics:** Specific, focused and personal. | **Topic Area:** Professionalism | **Intended Outcome:** Create meaning and build knowledge; Promote Reflection |
|  | **Type of Article:** Innovation article/Case report | **Aspects:** Reflections of experiences- where they have been, where they are and where they could be going | **Participant Characteristics:**  Interdisciplinary (Law, medical and Nursing Students) | **Type of doctor:** Various, non-specific |
|  | **Location:** Canada |  |  |  |
| Female doctors’ life narratives foster students’ self-reﬂection^61^ | **Author:** Y Lin | **Terminology Used:** Life narrative, life story | **Setting:** Not Stated | **Format:** Live spoken, in-person |
|  | **Year:** 2013 | **Definition:** None given | **Attendance Requirement:**  Voluntary | **Associated Elements:**  Reflection |
|  | **Journal:** Medical Education | **Characteristics:** Not stated | **Topic Area:** Reflection | **Intended Outcome:** Promote self-care, compassion and wellbeing; Promote Reflection |
|  | **Type of Article:** Innovation article/Case report | **Aspects:** A perspective on life and careers | **Participant Characteristics:**  Not Stated | **Type of doctor:** Female doctors |
|  | **Location:** Taiwan |  |  |  |
| Harnessing the medical humanities for experiential learning^34^ | **Author:** S Singh, et al. | **Terminology Used:** Narrative | **Setting:** Medical School | **Format:** Written prose and graphics |
|  | **Year:** 2017 | **Definition:** None given | **Attendance Requirement:** Voluntary | **Associated Elements:** Reflection |
|  | **Journal:** Indian Journal of Medical Ethics | **Characteristics:** Not stated | **Topic Area:** Medical Humanities | **Intended Outcome:** Enhance Professional Development; Enhance cultural, social and ethical awareness |
|  | **Type of Article:** Innovation article/Case report | **Aspects:** Not stated | **Participant Characteristics:** Mixed (medical students, interns and faculty members) | **Type of doctor:** Various, non-specific |
|  | **Location:** India |  |  |  |
| How medical teachers use narratives in lectures: a qualitative study^35^ | **Author:** G Easton | **Terminology Used:** Narrative, story, professional autobiography | **Setting:** Medical School | **Format:** Live spoken, in-person |
|  | **Year:** 2016 | **Definition:** None given | **Attendance Requirement:**  Compulsory | **Associated Elements:** Not stated |
|  | **Journal:** BMC Medical Education | **Characteristics:** Believable, memorable and engaging. | **Topic Area:** Narrative Medicine | **Intended Outcome:** Create meaning and build knowledge; Promote reflection; Promote Professional Identity Formation |
|  | **Type of Article:** Research Article | **Aspects:** Information about their own professional career | **Participant Characteristics:**  First year medical students | **Type of doctor:** Various, non-specific |
|  | **Location:** UK |  |  |  |
| Human Contexts: Medicine in Society at Stony Brook University School of Medicine^62^ | **Author:** J Coulehan et al. | **Terminology Used:** Narrative | **Setting:** Medical School | **Format:** Not stated |
|  | **Year:** 2003 | **Definition:** None given | **Attendance Requirement:**  Compulsory | **Associated Elements:** Group Discussion |
|  | **Journal:** Academic Medicine | **Characteristics:** Not stated | **Topic Area:** Medical Humanities | **Intended Outcome:** Enhance Professional Development; Promote Professional Identity Formation |
|  | **Type of Article:** Innovation article/Case report | **Aspects:** Not stated | **Participant Characteristics:**  Throughout the medical course (Years 1-5) | **Type of doctor:**  Not Stated |
|  | **Location:** USA |  |  |  |
| Incorporating Storytelling into the Medical Curriculum^21^ | **Author:** S Shirvastava et al. | **Terminology Used:** Storytelling, story, narrative, anecdote, real-life experience | **Setting:** Not stated | **Format:** Not stated |
|  | **Year:** 2023 | **Definition:** "the employment of narratives, anecdotes, and real-life experiences to stories to share and teach medical concepts and principles." | **Attendance Requirement:**  Not stated | **Associated Elements:**  Reflection |
|  | **Journal:** National Journal of Clinical Anatomy | **Characteristics:** Not stated | **Topic Area:** Narrative Medicine | **Intended Outcome:** Promote a better understanding of the human side of healthcare; Promote Reflection; Enhance cultural, social and ethical awareness |
|  | **Type of Article:** Commentary/Review Article | **Aspects:** Personal experiences. May also contain cultural scenarios and ethical situations. | **Participant Characteristics:**  Not stated | **Type of doctor:** Not Stated |
|  | **Location:** Not Stated |  |  |  |
| Incorporating the Arts and Humanities in Palliative Medicine Education^63^ | **Author:** L Marchand | **Terminology Used:** Story, narrative | **Setting:** Clinical Setting | **Format:** Live spoken, in-person |
|  | **Year:** 2006 | **Definition:** None given | **Attendance Requirement:**  Not Stated | **Associated Elements:** Group Discussion |
|  | **Journal:** Journal of Learning through the Arts | **Characteristics:** Not stated | **Topic Area:** Specialty specific- Palliative Medicine | **Intended Outcome:** Enhance Professional Development |
|  | **Type of Article:** Commentary/Review Article | **Aspects:** Not stated | **Participant Characteristics:** Mixed (Resident physicians, Junior Physicians, Medical Students, other Health Professionals) | **Type of doctor:** Various |
|  | **Location:** USA |  |  |  |
| Introducing compassion into the education of health care professionals; can Schwartz Rounds help?^36^ | **Author:** R Barker, J Cornwell and F Gishen | **Terminology Used:** Story, experience | **Setting:** Medical School | **Format:** Live spoken, in-person |
|  | **Year:** 2016 | **Definition:** None given | **Attendance Requirement:** Voluntary | **Associated Elements:** Group Discussion |
|  | **Journal:** Journal of Compassionate Healthcare | **Characteristics:** Not stated | **Topic Area:** Wellbeing- burnout, resilience and compassion | **Intended Outcome:** Promote self-care, compassion and wellbeing; Create a sense of community |
|  | **Type of Article:** Commentary/Review Article | **Aspects:** Not stated | **Participant Characteristics:** Fifth and sixth year medical students | **Type of doctor:** Various (including other healthcare staff) |
|  | **Location:** UK |  |  |  |
| Learner Experiences of Preceptor Self-Disclosure of Personal Illness in Medical Education^56^ | **Author:** I Cezara Erne, E Kocaqi, A Acai | **Terminology Used:** Lived experience, story, personal illness story, narrative | **Setting:** Medical School | **Format:** Not stated |
|  | **Year:** 2024 | **Definition:** None given | **Attendance Requirement:** Voluntary | **Associated Elements:** Not stated |
|  | **Journal:** Academic Medicine | **Characteristics:** Not stated | **Topic Area:** Wellbeing- Mental health | **Intended Outcome:** Create a sense of community; Promote self-care, compassion and wellbeing; Promote Professional Identity Formation |
|  | **Type of Article:** Research Article | **Aspects:** Not stated | **Participant Characteristics:** Medical Students (non-specific) | **Type of doctor:** Not stated |
|  | **Location:** Canada |  |  |  |
| Lewis Katz School of Medicine: Medical Humanities and the Narrative Medicine Program^37^ | **Author:** M Vitez and N Rosenberg | **Terminology Used:** Story, storytelling, narrative | **Setting:** External location | **Format:** Live spoken, in-person |
|  | **Year:** 2024 | **Definition:** None given | **Attendance Requirement:** Voluntary | **Associated Elements:**  Not Stated |
|  | **Journal:** Lewis Katz School of Medicine Website | **Characteristics:** Not stated | **Topic Area:** Narrative Medicine | **Intended Outcome:** Promote reflection; Create a sense of community; Promote a better understanding of the human side of healthcare |
|  | **Type of Article:** Other (Webpage) | **Aspects:** Not stated | **Participant Characteristics:**  Not stated | **Type of doctor:** Various, non-specific |
|  | **Location:** USA |  |  |  |
| Literary Inquiry and Professional Development in Medicine: Against Abstractions^72^ | **Author:** D Wear, L Nixon | **Terminology Used:** Narrative | **Setting:** Not stated | **Format:** Written prose and poetry |
|  | **Year:** 2002 | **Definition:** None given | **Attendance Requirement:**  Not stated | **Associated Elements:** Not stated |
|  | **Journal:** Perspectives in Biology and Medicine | **Characteristics:** Subjective | **Topic Area:** Professional Identity Formation; Professionalism | **Intended Outcome:** Enhance Professional Development |
|  | **Type of Article:** Commentary/Review Article | **Aspects:** May include experiences readers may have not lived, daily challenges of medicine. | **Participant Characteristics:**  Not stated | **Type of doctor:** Various, non-specific |
|  | **Location:** USA |  |  |  |
| Making space for stories: promoting physician and medical student well-being through successful medical education storytelling events^57^ | **Author:** M Olsen, B Trappey | **Terminology Used:** Story, storytelling, narrative | **Setting:** Online, Medical School | **Format:** Not stated |
|  | **Year:** 2024 | **Definition:** None given | **Attendance Requirement:**  Voluntary | **Associated Elements:** Reflection, sharing own story |
|  | **Journal:** BMC Medical Education | **Characteristics:** Not stated | **Topic Area:** Wellbeing – burnout, resilience and compassion | **Intended Outcome:** Create a sense of community; Promote self-care, compassion and wellbeing |
|  | **Type of Article:** Commentary/Review Article | **Aspects:** Not stated. | **Participant Characteristics:**  Medical Students (non-specific) and medical school faculty | **Type of doctor:** Various, non-specific |
|  | **Location:** USA |  |  |  |
| Narrative in medical ethics^73^ | **Author:** A Hudson Jones | **Terminology Used:** Narrative, story | **Setting:** Not stated | **Format:** Written prose |
|  | **Year:** 1999 | **Definition:** None given | **Attendance Requirement:**  Not stated | **Associated Elements:**  Not Stated |
|  | **Journal:** British Medical Journal (BMJ) | **Characteristics:** Not stated | **Topic Area:** Medic Ethics | **Intended Outcome:** Enhance cultural, social and ethical awareness; Promote self-care, compassion and wellbeing |
|  | **Type of Article:** Commentary/Review Article | **Aspects:** Not stated | **Participant Characteristics:**  Not stated | **Type of doctor:** Various, non-specific |
|  | **Location:** USA |  |  |  |
| Narrative medicine as a means of training medical students toward residency competencies^64^ | **Author:** S Arntfield et al. | **Terminology Used:** Narrative, doctors' account of practice | **Setting:** Medical School | **Format:** Written prose and poetry |
|  | **Year:** 2013 | **Definition:** None given | **Attendance Requirement:** Voluntary | **Associated Elements:**  Group Discussion; Reflection |
|  | **Journal:** Patient Education and Counselling | **Characteristics:** Not stated | **Topic Area:** Narrative Medicine | **Intended Outcome:** Enhance Professional Development; Create a sense of community |
|  | **Type of Article:** Research Article | **Aspects:** Not stated | **Participant Characteristics:**  Fourth year medical students | **Type of doctor:** Various, non-specific |
|  | **Location:** USA |  |  |  |
| Narrative Texts and Issues in Medical Humanities^38^ | **Author:** S van den Berg | **Terminology Used:** Narrative | **Setting:** Not stated | **Format:** Written prose and poetry |
|  | **Year:** 2015 | **Definition:** None given | **Attendance Requirement:** Not stated | **Associated Elements:**  Not Stated |
|  | **Journal:** Literature Compass | **Characteristics:** Not stated | **Topic Area:** Medical Humanities | **Intended Outcome:**  Promote reflection; Promote Professional Identity Formation |
|  | **Type of Article:** Commentary/Review Article | **Aspects:** Personal and professional experience. May also include professional dilemmas, struggles and insights of medical practice and the medical experience of decision making. | **Participant Characteristics:** Not stated | **Type of doctor:** Various- also includes patient, caregivers and other healthcare staff |
|  | **Location:** USA |  |  |  |
| Perspectives about Professionalism among Undergraduate Students in a Medical College in India: A Qualitative Study^39^ | **Author:** P T Dhikale, S R Shrivastava, S Srinivasan. | **Terminology Used:** Narratives, case study, case vignette, experience | **Setting:** Not stated | **Format:** Not stated |
|  | **Year:** 2020 | **Definition:** None given | **Attendance Requirement:**  Not stated | **Associated Elements:** Not stated |
|  | **Journal:** Indian Journal of Community Medicine | **Characteristics:** Not stated | **Topic Area:** Professionalism | **Intended Outcome:** Enhance Professional Development |
|  | **Type of Article:** Research Article | **Aspects:** Not stated | **Participant Characteristics:**  Not stated | **Type of doctor:** Not Stated |
|  | **Location:** India |  |  |  |
| Physician Self-disclosure of Lived Experience Improves Mental Health Attitudes Among Medical Students: A Randomized Study^40^ | **Author:** A Martin et al. | **Terminology Used:** Self-disclosed history, lived experience | **Setting:** Medical School | **Format:** Live spoken, in-person |
|  | **Year:** 2020 | **Definition:** None given | **Attendance Requirement:**  Not Stated | **Associated Elements:**  Group Discussion |
|  | **Journal:** Journal of Medical Education and Curricular Development | **Characteristics:** Not stated | **Topic Area:** Wellbeing- Mental Health | **Intended Outcome:** Change attitudes, perceptions and beliefs |
|  | **Type of Article:** Research Article | **Aspects:** Not stated | **Participant Characteristics:**  Second year medical students | **Type of doctor:** Doctors who had experience of living with a mental health condition or had previously accessed mental health services |
|  | **Location:** Israel |  |  |  |
| Professional Stigma of Mental Health Issues: Physicians Are Both the Cause and Solution^41^ | **Author:** K Brower | **Terminology Used:** Story | **Setting:** Not stated | **Format:**  Not Stated |
|  | **Year:** 2021 | **Definition:** None given | **Attendance Requirement:** Not stated | **Associated Elements:** Not Stated |
|  | **Journal:** Academic Medicine | **Characteristics:** Not stated | **Topic Area:** Wellbeing- Mental Health | **Intended Outcome:** Change attitudes, perceptions and beliefs |
|  | **Type of Article:** Commentary/Review Article | **Aspects:** Not stated | **Participant Characteristics:**  Not stated | **Type of doctor:** Not Stated |
|  | **Location:** USA |  |  |  |
| REACH: A Required Curriculum to Foster the Well-Being of Medical Students^42^ | **Author:** C Ferguson, T Ark and A Kalet | **Terminology Used:** Personal story; storytelling | **Setting:** Medical School | **Format:** Live spoken, in-person |
|  | **Year:** 2022 | **Definition:** None given | **Attendance Requirement:**  Compulsory | **Associated Elements:** Reflection; Sharing own story |
|  | **Journal:** Academic Medicine | **Characteristics:** Not stated | **Topic Area:** Wellbeing | **Intended Outcome:** Enhance Professional Development; Promote Self-care, compassion and wellbeing |
|  | **Type of Article:** Innovation article/Case report | **Aspects:** Not stated | **Participant Characteristics:** First and second year medical students | **Type of doctor:** Faculty members (some of whom are doctors) |
|  | **Location:** USA |  |  |  |
| Rediscovering Empathy in Medical Education: Experiencing Literature and Film^65^ | **Author:** E Beckman | **Terminology Used:** Narrative, story | **Setting:** Not stated | **Format:** Written Prose |
|  | **Year:** 2006 | **Definition:** None given | **Attendance Requirement:** Not stated | **Associated Elements:**  Discussion; Reflection |
|  | **Journal:** Dissertation Abstracts International | **Characteristics:** Not stated | **Topic Area:** Empathy | **Intended Outcome:**  Promote a better understanding of the human side of healthcare; Promote Reflection; Enhance Professional Development |
|  | **Type of Article:** Other (PhD Thesis) | **Aspects:** Not stated | **Participant Characteristics:** Not stated | **Type of doctor:** Not Stated |
|  | **Location:** USA |  |  |  |
| Reducing mental health stigma in medical students and doctors towards their peers with mental health difficulties: a protocol^43^ | **Author:** A Hankir et al. | **Terminology Used:** Lived experience; personal testimony | **Setting:** Medical School | **Format:** Live spoken, in-person |
|  | **Year:** 2020 | **Definition:** None given | **Attendance Requirement:** Voluntary | **Associated Elements:**  Not Stated |
|  | **Journal:** Psychiatria Dambina | **Characteristics:** Not stated | **Topic Area:** Wellbeing- Mental Health | **Intended Outcome:** Change attitudes, perceptions and beliefs |
|  | **Type of Article:** Other (Conference Paper) | **Aspects:** Not stated | **Participant Characteristics:**  Medical Students recruited through local student psychiatry societies in the UK | **Type of doctor:** Not Stated |
|  | **Location:** UK |  |  |  |
| Schwartz Centre Rounds: a new initiative in the undergraduate curriculum—what do medical students think?^44^ | **Author:** F Gishen et al. | **Terminology Used:** Cases; story | **Setting:** Medical School | **Format:** Live spoken, in-person |
|  | **Year:** 2016 | **Definition:** None given | **Attendance Requirement:** Voluntary | **Associated Elements:**  Group Discussion |
|  | **Journal:** BMC Medical Education | **Characteristics:** Not stated | **Topic Area:** Reflection | **Intended Outcome:** Promote reflection; Enhance professional development |
|  | **Type of Article:** Research Article | **Aspects:** Not stated | **Participant Characteristics:**  Fifth year medical students | **Type of doctor:** Various including Medical students and Nurses |
|  | **Location:** UK |  |  |  |
| Schwartz rounds in undergraduate medical education facilitates active reflection and individual identification of learning need^45^ | **Author:** C Stocker et al. | **Terminology Used:** Personal experience; story; anecdotes | **Setting:** Medical School | **Format:** Live spoken, in-person |
|  | **Year:** 2018 | **Definition:** None given | **Attendance Requirement:**  Not Stated | **Associated Elements:**  Group Discussion |
|  | **Journal:** MedEdPublish | **Characteristics:** Not stated | **Topic Area:** Wellbeing; Professionalism | **Intended Outcome:** Promote reflection |
|  | **Type of Article:** Research Article | **Aspects:** Personal experiences and reflections of the psychological aspects of caring. | **Participant Characteristics:**  Second year medical students | **Type of doctor:** Various, non-specific |
|  | **Location:** UK |  |  |  |
| Shared Living Experiences by Physicians have a Positive Impact on Mental Health Attitudes and Stigma among Medical Students: A Mixed-Methods Study^46^ | **Author:** A Martin et al. | **Terminology Used:** Living experience; history | **Setting:** Medical School | **Format:** Live, spoken, online |
|  | **Year:** 2020 | **Definition:** None given | **Attendance Requirement:**  Compulsory | **Associated Elements:**  Group Discussion |
|  | **Journal:** Journal of Medical Education and Curricular Development | **Characteristics:** Not stated | **Topic Area:** Wellbeing- Mental Health | **Intended Outcome:** Change attitudes, perceptions and beliefs |
|  | **Type of Article:** Research Article | **Aspects:** Not stated | **Participant Characteristics:**  Second year medical students | **Type of doctor:** Senior doctors |
|  | **Location:** Israel |  |  |  |
| Storytelling as a Method for Teaching Values and Attitudes^74^ | **Author:** W Hensel and T Rasco | **Terminology Used:** Story, storytelling, life story | **Setting:** Not stated | **Format:** Live spoken, in-person |
|  | **Year:** 1992 | **Definition:** "the big things… of the physician's life- the great unmentionables that are yet everyday aspects of doctoring" | **Attendance Requirement:**  Not stated | **Associated Elements:**  Discussion, reflection |
|  | **Journal:** Academic Medicine | **Characteristics:** Personal, well-focused and derived from the immediate clinical setting. | **Topic Area:** Professionalism | **Intended Outcome:** Promote reflection; Enhance Professional development |
|  | **Type of Article:** Commentary/Review Article | **Aspects:** Have an important specific message. Right amount of information (to promote reflection and discussion). | **Participant Characteristics:**  Not stated | **Type of doctor:** Various, non-specific |
|  | **Location:** USA |  |  |  |
| Teaching leadership: the medical student society model^47^ | **Author:** J Matthews et al. | **Terminology Used:** Personal story; storytelling | **Setting:** Not Stated | **Format:** Not Stated |
|  | **Year:** 2018 | **Definition:** None given | **Attendance Requirement:**  Voluntary | **Associated Elements:**  Not Stated |
|  | **Journal:** The Clinical Teacher | **Characteristics:** Not stated | **Topic Area:** Leadership and Management | **Intended Outcome:**Create meaning and build knowledge |
|  | **Type of Article:** Innovation article/Case report | **Aspects:** Personal Experience | **Participant Characteristics:**  Not Stated | **Type of doctor:** Doctors and others who work in a leadership or management role in the NHS |
|  | **Location:** UK |  |  |  |
| The introduction of medical humanities in the undergraduate curriculum of Greek medical schools: challenge and necessity^66^ | **Author:** A Batistatou et al. | **Terminology Used:** Narrative, story, biography | **Setting:** Medical School | **Format:** Not Stated |
|  | **Year:** 2010 | **Definition:** None given | **Attendance Requirement:** Not Stated | **Associated Elements:**  Not Stated |
|  | **Journal:** Hippokratia | **Characteristics:** Inspiring, Role-model generating | **Topic Area:** Medical Humanities | **Intended Outcome:** Promote a better understanding of the human side of healthcare |
|  | **Type of Article:** Commentary/Review Article | **Aspects:** Not stated | **Participant Characteristics:**  Not Stated | **Type of doctor:** Not Stated |
|  | **Location:** Greece |  |  |  |
| The Study of Literature in Medical Education^75^ | **Author:** K Hunter, R Charon and J Coulehan | **Terminology Used:** Narrative, story | **Setting:** Not stated | **Format:** Not Stated |
|  | **Year:** 1995 | **Definition:** None given | **Attendance Requirement:** Not stated | **Associated Elements:** Not Stated |
|  | **Journal:** Academic Medicine | **Characteristics:** Not stated | **Topic Area:** Narrative Medicine | **Intended Outcome:** Promote reflection; Enhance professional development |
|  | **Type of Article:** Commentary/Review Article | **Aspects:** Not stated | **Participant Characteristics:** Not stated | **Type of doctor:** Not Stated |
|  | **Location:** USA |  |  | **Type of doctor:** Not Stated |
| Things we are expected to just do and deal with’: Using the medical humanities to encourage reﬂection on vulnerability and nurture clinical skills, collegiality, compassion, and selfcare^48^ | **Author:** M Kelly et al. | **Terminology Used:** Story, narrative | **Setting:** Not Stated | **Format:** Live spoken, in-person; written prose |
|  | **Year:** 2022 | **Definition:** None given | **Attendance Requirement:**  Compulsory | **Associated Elements:**  Reflection |
|  | **Journal:** Perspect Medical Education | **Characteristics:** Not stated | **Topic Area:** Medical Humanities | **Intended Outcome:** Promote reflection; Promote self-care, compassion and wellbeing |
|  | **Type of Article:** Innovation article/Case report | **Aspects:** Not stated | **Participant Characteristics:**  Not Stated | **Type of doctor**: "Generalist" doctors (from geriatrics, rehabilitation or palliative medicine). |
|  | **Location:** Australia |  |  |  |
| Virtues Education in Medical School: The Foundation for Professional Formation^49^ | **Author:** L Seoane et al | **Terminology Used:** Narrative | **Setting:** Medical School | **Format:** Live spoken, in-person |
|  | **Year:** 2016 | **Definition:** None given | **Attendance Requirement:**  Compulsory | **Associated Elements:**  Reflection |
|  | **Journal:** Ochsner Journal | **Characteristics:** Not stated | **Topic Area:** Professional Identity Formation | **Intended Outcome:** Promote Professional Identity Formation |
|  | **Type of Article:** Innovation article/Case report | **Aspects:** Not stated | **Participant Characteristics:**  Fourth year medical students | **Type of doctor:** Not Stated |
|  | **Location:** Australia |  |  |  |
| Wanted: role models - medical students’ perceptions of professionalism^67^ | **Author:** A Byszewski et al. | **Terminology Used:** Reflective narrative, real case | **Setting:** Not stated | **Format:** Live spoken, in-person |
|  | **Year:** 2012 | **Definition:** None given | **Attendance Requirement:**  Not stated | **Associated Elements:** Not Stated |
|  | **Journal:** BMC Medical Education | **Characteristics:** Not stated | **Topic Area:** Professionalism | **Intended Outcome:** Enhance Professional Development |
|  | **Type of Article:** Research Article | **Aspects:** Not stated | **Participant Characteristics:**  Not stated | **Type of doctor:** Not Stated |
|  | **Location:** Canada |  |  |  |
| What About Empathy? A Qualitative Study Exploring the Role of a Podcast as an Asynchronous Empathy Teaching Tool^59^ | **Author:** I Ying et al. | **Terminology Used:** Story, storytelling | **Setting:** Online | **Format:** Recorded spoken audio |
|  | **Year:** 2024 | **Definition:** None given | **Attendance Requirement:**  Voluntary | **Associated Elements:** Not Stated |
|  | **Journal:** Medical Science Educator | **Characteristics:** Not stated | **Topic Area:** Empathy | **Intended Outcome:** Create a sense of community; Promote reflection; Change attitudes, perceptions and beliefs; Enhance professional development; Promote a better understanding of the human side of healthcare; Create meaning and build knowledge |
|  | **Type of Article:** Research Article | **Aspects:** Not stated | **Participant Characteristics:**  Mixed with medical students, other healthcare students and qualified healthcare professionals | **Type of doctor:** Not Stated |
|  | **Location:** Canada |  |  |  |
| What Students Learn About Professionalism From Faculty Stories: An “Appreciative Inquiry” Approach^68^ | **Author:** J Quaintance, L Arnold and G Thompson | **Terminology Used:** Narrative, story, narrative storytelling | **Setting:** Medical School | **Format:** Live spoken, in-person |
|  | **Year:** 2010 | **Definition:** None given | **Attendance Requirement:**  Not Stated | **Associated Elements:**  Reflection; Sharing own story |
|  | **Journal:** Academic Medicine | **Characteristics:** Not stated | **Topic Area:** Professionalism | **Intended Outcome:** Promote reflection; Enhance professional development |
|  | **Type of Article:** Research Article | **Aspects:** Not stated | **Participant Characteristics:**  All years of medical students | **Type of doctor:** Internists (doctors who work in the Intensive Care Unit) |
|  | **Location:** USA |  |  |  |
| What Will You Protect? Redeﬁning Professionalism Through the Lens of Diverse Personal Identities^50^ | **Author:** A Bhatia-lin et al. | **Terminology Used:** Experience, shared physician narrative | **Setting:** Medical School | **Format:** Live spoken, in-person and live spoken, online |
|  | **Year:** 2021 | **Definition:** None given | **Attendance Requirement:**  Compulsory | **Associated Elements:**  Discussion; Reflection |
|  | **Journal:** The AAMC Journal of Teaching and Learning Resources | **Characteristics:** Not stated | **Topic Area:** Professional Identity Formation; Professionalism | **Intended Outcome:** Promote reflection; Promote Professional Identity Formation |
|  | **Type of Article:** Innovation article/Case report | **Aspects:** Doctors' own experiences | **Participant Characteristics:**  First year medical students | **Type of doctor:** Not Stated |
|  | **Location:** USA |  |  |  |
| When Bad Things Happen: Training Medical Students to Anticipate the Aftermath of Medical Errors^51^ | **Author:** S Musnur et al. | **Terminology Used:** Narrative, personal anecdote, personal story | **Setting:** Medical School | **Format:** Live spoken, in-person and recorded spoken video |
|  | **Year:** 2020 | **Definition:** None given | **Attendance Requirement:**  Compulsory | **Associated Elements:** Reflection; Sharing own story |
|  | **Journal:** Academic Psychiatry | **Characteristics:** Not stated | **Topic Area:** Wellbeing; Patient safety- medical errors | **Intended Outcome:** Enhance Professional Development |
|  | **Type of Article:** Innovation article/Case report | **Aspects:** Reflections on clinical experiences | **Participant Characteristics:**  Second year medical students | **Type of doctor:** Various, non-specific |
|  | **Location:** USA |  |  |  |
| Written role models in professionalism education^69^ | **Author:** J Coulehan | **Terminology Used:** Narrative, personal story | **Setting:** Not stated | **Format:** Written Prose |
|  | **Year:** 2007 | **Definition:** None given | **Attendance Requirement:**  Not stated | **Associated Elements:**  Not Stated |
|  | **Journal:** Medical Humanities | **Characteristics:** Not stated | **Topic Area:** Professionalism | **Intended Outcome:** Promote reflection; Enhance professional development |
|  | **Type of Article:** Commentary/Review Article | **Aspects:** Values, beliefs and community | **Participant Characteristics:**  Not stated | **Type of doctor:** Various, non-specific |
|  | **Location:** USA |  |  |  |
| You’re Not Alone: Sharing of Anonymous Narratives to Destigmatize Mental Illness in Medical Students and Faculty^52^ | **Author:** R Pillai et al. | **Terminology Used:** Narrative, personal story | **Setting:** Medical School | **Format:** Live spoken, in-person |
|  | **Year:** 2020 | **Definition:** None given | **Attendance Requirement:** Voluntary | **Associated Elements:**  Not Stated |
|  | **Journal:** Academic Psychiatry | **Characteristics:** Not stated | **Topic Area:** Wellbeing- Mental Health | **Intended Outcome:** Create a sense of community; Change attitudes, perceptions and beliefs |
|  | **Type of Article:** Innovation article/Case report | **Aspects:** Not stated | **Participant Characteristics:**  Mixed (medical students, faculty, nursing students) | **Type of doctor:** Not stated |
|  | **Location:** USA |  |  |  |
