# Supplementary material for: Doctors’ life stories in undergraduate medical education: definition, key concepts and uses – a scoping review
Source: BMC Med Educ. 2025 Oct 9;25:1390. doi: 10.1186/s12909-025-07960-8 (PMC12512262; doi:10.1186/s12909-025-07960-8)
Supplement: Supplementary file 4 — Additional file4. Data Charting Guidance Form. Description of data: A table documenting the guidance used when carrying out data charting as part of this scoping review [file 12909_2025_7960_MOESM4_ESM.docx]

# Additional File 4: Data Charting Guidance Form

| Category | Sub-category | Description |
| --- | --- | --- |
| Publication Characteristics | Title | Title of the article |
|  | Author | Named authors of the article |
|  | Year of publication | Year article first published |
|  | Journal | Journal/Place where article published |
|  | Type of Article | What type of article is it (e.g. research, review, commentary etc.)? |
|  | Location | Where does the article originate from, what country? |
| Terminology | Terminology used | What terminology (or terms) were used within the article to describe doctors' life stories? |
|  | Definition | What is the definition of doctors' life stories (or the terminology used in the article) described within the article? |
|  | Characteristics | What important characteristics of doctors' life stories are described in the article? (i.e. descriptors of doctors' life stories) |
|  | Aspects | What important aspects of doctors' life stories are described in the article? (i.e. things/elements that make doctors' life stories) |
| Methods | Setting | In what setting were doctors' life stories used (or described)? |
|  | Attendance Requirement | What was the attendance requirement for participants? Was it voluntary sign up? Or was it compulsory as part of their course? |
|  | Topic Area | What topic area were doctors' life stories used in (e.g. professionalism etc.)? |
|  | Participant Characteristics | Who were the participants/potential participants? If medical students, what stage were the participants (e.g. first year, pre-clinical etc.)? |
| Intervention | Format | What format are doctors' life stories used in (e.g. online, live, spoken, written etc.) |
|  | Associated Elements | Were there any other elements used within the session alongside doctors’ life stories (e.g. discussion)? |
|  | Intended Outcome | What was the authors intended outcome for the use of doctors' life stories? |
|  | Type of doctor | Was there a description of the author (doctor) of the doctors' life story/stories. If so what detail did they provide (e.g. specialty, grade etc.) |
